# Genetic Characteristics of the Human Hepatic Stellate Cell Line LX-2

**DOI:** 10.1371/journal.pone.0075692

**Published:** 2013-10-08

**Authors:** Ralf Weiskirchen, Jörg Weimer, Steffen K. Meurer, Anja Kron, Barbara Seipel, Inga Vater, Norbert Arnold, Reiner Siebert, Lieming Xu, Scott L. Friedman, Carsten Bergmann

**Affiliations:** 1 Institute of Clinical Chemistry and Pathobiochemistry, RWTH Aachen University, Aachen, Germany; 2 Department of Gynaecology and Obstetrics, UKSH Campus Kiel, Kiel, Germany; 3 Center for Human Genetics, Bioscientia, Ingelheim, Germany; 4 Institute of Human Genetics, University Hospital Schleswig-Holstein & Christian-Albrechts University Kiel, Kiel, Germany; 5 Institute of Liver Diseases, Shanghai University of Traditional Chinese Medicine Shuguang Hospital, Shanghai, PR China; 6 Division of Liver Diseases, Mount Sinai School of Medicine, New York, New York, United States of America; 7 Department of Human Genetics, RWTH Aachen University, Aachen, Germany; 8 Center for Clinical Research, University Hospital Freiburg, Freiburg, Germany; University of Sydney, Australia

## Abstract

The human hepatic cell line LX-2 has been described as tool to study mechanisms of hepatic fibrogenesis and the testing of antifibrotic compounds. It was originally generated by immortalisation with the Simian Vacuolating Virus 40 (SV40) transforming (T) antigen and subsequent propagation in low serum conditions. Although this immortalized line is used in an increasing number of studies, detailed genetic characterisation has been lacking. We here have performed genetic characterisation of the LX-2 cell line and established a single-locus short tandem repeat (STR) profile for the cell line and characterized the LX-2 karyotype by several cytogenetic and molecular cytogenetic techniques. Spectral karyotyping (SKY) revealed a complex karyotype with a set of aberrations consistently present in the metaphases analyses which might serve as cytogenetic markers. In addition, various subclonal and single cell aberrations were detected. Our study provides criteria for genetic authentication of LX-2 and offers insights into the genotype changes which might underlie part of its phenotypic features.

## Introduction

During liver fibrogenesis and the establishment of cirrhosis, contractile myofibroblasts (MFB) that originate from quiescent hepatic stellate cells (HSC) are the major source of extracellular matrix (ECM) [1]. HSC/MFB possesses the capacity not only for matrix synthesis, but also for the expression and secretion of pro- and anti-inflammatory cytokines and growth factors [2]. Based on their pivotal role in the initiation and progression of liver fibrogenesis, HSC/MFB biology is a major focus of fibrosis investigation. However, the preparation of primary cells from the liver is time-consuming and requires special expertise. To overcome these limitations, several spontaneous or experimentally-derived immortalized HSC cell lines from mouse, rat and humans have been established [3]. Like other permanent cell lines, immortalized stellate cell lines have the advantage of growing continuously to provide unlimited access. Moreover, their clonal origin usually guarantees a considerably homogeneous phenotype that should allow the performance of reproducible experiments in different laboratories [3]. Based on this approach, key aspects of stellate cell biology have been uncovered including advances in retinoid metabolism, extracellular matrix expression and turnover, cytokine production and signalling, and gene regulation. In particular, these cell lines are exploited to develop therapeutic approaches. In this context, there is an increasing need for properly characterized stellate cell lines that preserve phenotypic characteristics of HSC/MFB especially for the human derivatives.

The human HSC cell lines Lieming Xu-1 (LX-1) and Lieming Xu-2 (LX-2) were originally generated by transformation of cultured primary HSC obtained from a male human liver with a plasmid encoding the SV40 large T-antigen expressed under the control of a Rous sarcoma virus promoter (LX-1) or by spontaneous immortalization of a subset of early passaged LX-1 cells that were grown in low serum conditions (LX-2) [4]. Both cell lines express α-SMA, vimentin, the intermediate filament protein glial fibrillary acidic protein (GFAP), and the type β receptor for platelet-derived growth (PDGFRβ) suggesting that both cell lines retain key features of activated/transdifferentiated HSC. Both LX cell lines also secrete pro-collagen, pro-MMP-2, MT1-MMP (MMP-14), TIMP-1 and TIMP-2, all features characteristic for activated HSC [5]. Based on these properties, both LX cell lines have been widely employed as experimental tools in many laboratories worldwide. In particular, LX-2 enjoys great popularity among researchers interested in the elucidation of mechanisms underlying stellate cell biology and liver fibrosis, which is reflected by the increasing number of publications in which this cell line was utilized. Since the first report in 2003 [6] and the more detailed characterisation two years later [4], LX-2 cells have been cited nowadays in 158 peer reviewed publications (Figure S1) not only in the field of gastroenterology and hepatology [7]–[9] but also in studies focused on cellular and molecular biology [10], pharmacology/toxicology [11], lipid metabolism [12], tissue engineering [13], oncology [14], endocrinology [15] and other general topics [16].

Although widely used, LX-2, like all other immortalized cell lines, might be prone to genotypic/karyotypic and phenotypic drifts due to repeated passaging, which may result in cellular sub-lines that are phenotypically and genetically heterogeneous in character.

As a consequence, key results obtained with these or other cell lines should be validated in primary cells if possible. Alternatively, further efforts should be made to understand the diverse and heterogeneous outcomes observed with cell lines by more sophisticated characterization of the respective line. With this in mind, we have characterized the genetic profile of LX-2 using a number of cytogenetic and molecular methods, including single-locus short tandem repeat (STR) genotyping, standard karyotyping, spectral karyotyping (SKY), and single nucleotide polymorphism (SNP) array analytics. Our findings indicate that LX-2 cells comprise a complex karyotype with various structural abnormalities. However, the invariant STR signature and defined specific translocations could be established for LX-2 cells. Our results will facilitate proper identity testing, could lead to further mechanistic insights and improve experimental data in the field of liver fibrosis using this cell line. The work also provides importance guidance for the standardized, rigorous evaluation of other cell lines used in experimental hepatology.

## Materials and Methods

### Cell Culture

Human hepatic stellate cell line LX-2 [4], Wi-38 (clone VA-13, subline 2RA) (#CCL-75.1, ATCC, Manassas, VA, USA) [17], HepG2 [18], and Hep3B [18] were cultured in HEPES-buffered Dulbecco's modified Eagle medium (DMEM) (Lonza BioWhittaker, Verviers, Belgium) supplemented with 4 mmol/L L-glutamine (Lonza), 100 IU/ml Penicillin/100 µg/ml Streptomycin (Lonza), and 10% (v/v) (Wi-38, HepG2) or 2% (v/v) (LX-2) fetal calf serum (FCS) (PAA Laboratories, Pasching, Austria). Human umbilical vein endothelial cells (HUVEC) were isolated from human umbilical cords by collagenase dissociation and grown medium 199 (Gibco, Life Technologies, Darmstadt, Germany) with 20% heat-inactivated fetal bovine serum (FBS) (Gibco), 100 µg/ml endothelial cell growth supplement (Sigma, Taufkirchen, Germany), and 50 U/ml heparin (Sigma). Human MFB (hMFB) were isolated as outgrowth from human liver tissues and sub-cultured for several passages as described before [19]. All experiments done with LX-2 cells were done with passages number between p5 and p7 in which these numbers refer to those after selection of an immortalized cell population [4].

### Immunocytochemistry

Approximately 3×10^5^ LX-2 cells were plated on coverslips mounted in 6-well dishes and incubated for 24 h at 37°C. Thereafter, the cells were fixed for 15 min in 4% (w/v) paraformaldehyde buffered in phosphate-buffered saline (PBS) (pH 7.4) and permeabilized (2 min on ice) in 0.1% (w/v) sodium citrate/0.1% (v/v) Triton X-100. Then the cells were incubated with a rabbit polyclonal antibody raised against amino acids 4–30 mapping near the N-terminus of SV40 T Ag of SV40 origin (sc-20800, Santa Cruz Biotechnology, Santa Cruz, CA), a monoclonal antibody raised against α-smooth muscle actin (α-SMA) or respective normal rabbit (sc-2027, Santa Cruz) or mouse (sc-2343) control IgGs (obtained from Santa Cruz). The antibodies were applied for 1 h at 37°C in 1% (w/v) bovine serum albumin in PBS followed by incubation with a goat anti-rabbit IgG-FITC conjugate (sc-2012, Santa Cruz). Cells were mounted in antifade reagent and analysed in UV light using a standard objective. Filamentous actin of LX-2 cells was stained with the Alexa Fluor® 488 phalloidin conjugate (A12379, Molecular Probes, Invitrogen, Darmstadt, Germany). Nuclei were counterstained with 4′,6-diamidino-2-phenylindole (DAPI).

### Western Blot Analysis

Protein extracts were prepared from primary hMFB, LX-2, HepG2, Hep3B, Wi-38, HepG2 and HUVEC cells following standard protocols. Equal amounts of proteins were heated at 80°C for 10 min and separated in 4–12% Bis-Tris gels (Invitrogen) under reducing conditions and electro-blotted on nitrocellulose membranes (Schleicher & Schuell, Dassel, Germany). Equal protein loading was monitored in Ponceau S stain and unspecific binding sites were blocked in TBST [10 mM Tris/HCl, 150 mM NaCl, 0.1% (v/v) Tween 20, (pH 7.6)] containing 5% (w/v) non-fat milk powder. The membranes were subsequently probed with the antibodies given in Table S1. Primary antibodies were detected with horseradish-peroxidase (HRP)-conjugated secondary antibodies (Santa Cruz) and the Supersignal™ chemiluminescent substrate (Perbio Science, Bonn, Germany).

### G-banding, SKY and STR Analysis

LX-2 cells were trypsinized using Trypsin/EDTA and transferred in multiple T25 flasks and cultured for four days. The flask cultures were harvested following standard protocols including treatment with colcemid (10 µg/mL), hypotonic KCl solution (0.4%) and fixative (methanol: acetic acid = 3∶1). G-banding by Pancreatin staining with Giemsa (GPG) was performed following standard procedures. For conventional chromosome analysis, five cells were analyzed by light microscopy and karyotyped using the CytoVision Software (Leica Microsystems). With this method an average banding resolution of 250 bands was achieved. Aberrations were described according to the International Standard of Cytogenetic Nomenclature (ISCN 2013) [20]. For SKY analysis, metaphases were hybridized with the 24-color SKY Paint kit (ASI Applied Spectral Imaging Ltd., Migdal HaEmek, Israel) according essentially to the manufacturer’s protocol. Spectral images of hybridized metaphases were acquired using the SkyVision II Cytogenetic workstation and analyzed with the SkyView software version 1.6 (ASI). In addition, images of the DAPI counter-stain of the respective metaphases were acquired as regular CCD images using the direct mode of the SkyVision II system. Inversed and band-enhanced DAPI images were used for the intrachromosomal localization of aberrant chromosome areas. Data from the SKY analysis of LX-2 cells was submitted to the public SKY/M-Fish & CGH database platform of the National Cancer Institute (http://www.ncbi.nlm.nih.gov/sky/) that was established for investigators to share and compare their molecular cytogenetic data.

For STR multiplex analysis of LX-2 cells, two different multiplex assays were taken. The AmpFlSTR Identifiler® PCR amplification kit that amplifies 15 tetranucleotide repeat loci and the Amelogenin gender-determining marker was obtained from Life Technologies. The PowerPlex® 1.2 System that detects nine loci (eight STR loci and Amelogenin) was obtained from Promega (Mannheim, Germany). STR matching analysis of the obtained STR profile for LX-2 cells was done using the online STR analysis provided by the DSMZ (http://www.dsmz.de) and the ATCC STR Profile database for human cell lines (www.atcc.org) that actually (April 5, 2013) contains 2455 (DSMZ) or 1521 (ATCC) human PowerPlex® 1.2. system-genotyped cell lines. The STR profile of LX-2 cells was submitted and deposited in the DSMZ depository.

### Microarray Analysis

The Genome-Wide Human SNP Array 6.0 (Affymetrix, Santa Clara, CA) has been used according to the protocol provided by the manufacturer (http://www.affymetrix.com). Microarrays were washed and stained with the Fluidics Station 450 (Affymetrix) and scanned with the GeneChip Scanner 3000 (Affymetrix) using the Genotyping Console software version 4.1 (Affymetrix). The Birdseed v2 algorithm was used for genotyping. Copy number analysis, loss of heterozygosity (LOH) analysis and segmentation were calculated using Genotyping Console software version 4.1. Segments with aberrant copy number were considered as copy number aberration only if they consisted of at least 20 consecutive SNPs and comprised a minimal size of 100 kb.

## Results and Discussion

### LX-2 Cells Express the SV40 Large T-antigen and Markers Characteristic for Activated/transdifferentiated HSC

LX-2 cells were first introduced in 2003 [6] and initially characterized in 2005 [4] as a low-passaged human cell line derived from normal human stellate cells that are spontaneously immortalized. Cells were originally selected by their ability to grow under low serum conditions. The first preliminary description revealed that LX-2 cells exhibit typical markers of stellate cells, which in primary culture express desmin and GFAP. Moreover, LX-2 cells are responsive to platelet-derived growth factor BB and transforming growth factor-β1, which are the most prominent mitogenic (PDGF-BB) or pro-fibrogenic (TGF-β) stimuli towards primary stellate cells, respectively. The expression of α-SMA under all culture conditions indicates that LX-2 cells have a partially activated phenotype [4]. This conclusion is further supported by their fibroblast-like shape when grown to higher density and their somewhat star-shaped phenotype at lower cell numbers (Figure S2), as well as their high proliferative activity in culture (data not shown). In addition, when early passages of LX-2 cells were stained with fluorescently-labeled phalloidin that binds F-actin with high selectivity and affinity, all cells analysed showed a uniform staining pattern with some preferential expression in distinct areas of the cell membrane and around the nucleus (Figure S3). A similar staining pattern was observed when cells were stained with an antibody directed against α-SMA, confirming their mesenchymal and contractile (i.e., smooth muscle cell) origin (Figure S3). In the same set of experiments, we further stained early passages of LX-2 cells for SV40 T Ag that was used for immortalization and found that the nuclei of all cells stained positive for this viral oncogene product demonstrating that all cells within the analysed LX-2 culture are more or less homogeneous and further indicates that the T Ag is stably integrated into the genome (Figure S3).

**Figure 1:**
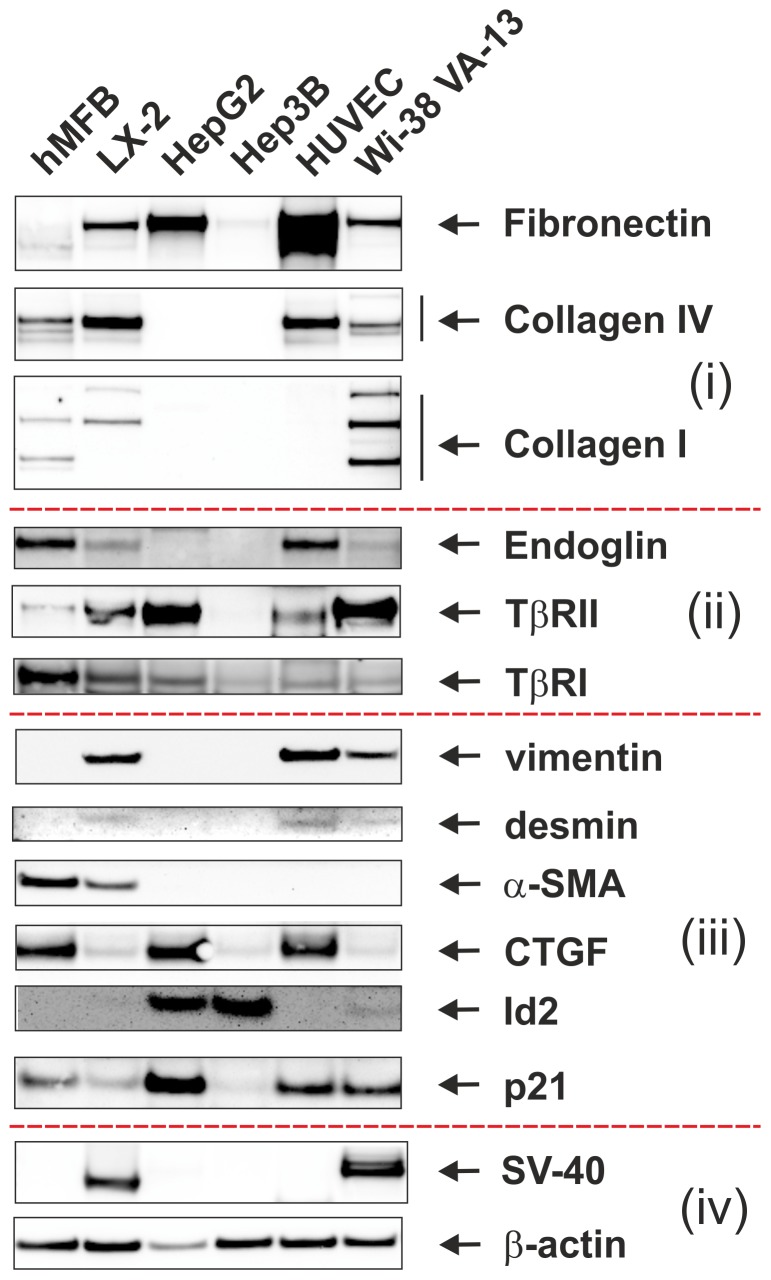
Expression analysis. Cell extracts were prepared from hMFB, LX-2, HepG2, Hep3B, HUVEC and Wi-38 VA-13 subline 2RA and analysed in Western blot for expression of (i) extracellular matrix proteins (fibronectin, collagen IV, collagen I), (ii) receptors of TGF-β (endoglin, TβRII, TβRI), (iii) HSC/MFB markers (vimentin, desmin, α-SMA, CTGF, Id2, p21), and (iv) SV40 large T-antigen and β-actin. We used two parenchymatic (HepG2, Hep3B) and other cell entities (hMFB, HUVEC, and WI-38) as controls because they are well characterized for the expression of tested genes and served as positive or negative controls in this analysis.

The expression of the SV40 large T-antigen was also confirmed in Western blot analysis using a rabbit polyclonal antibody raised against amino acids 4–30 mapping near the N-terminus of SV40 T Ag. In this analysis, we used extracts from Wi-38 cells as a positive control representing a SV40- transformed human diploid cell line derived from embryonic lung tissue of a female Caucasian that express both the SV40 neo (T) antigen and the SV40 transplantation antigen [21], [22]. In line with our immunocytochemical analysis, the Western blot revealed that LX-2 cells express low levels of SV40 T-Ag (Figure 1), while the two human hepatoma-derived cell lines HepG2 and Hep3B as well as HUVEC cells were negative for this oncogenic gene product.

LX-2 cells also express fibronectin, collagen type IV, collagen type I, endoglin, TGF-β receptors type II (TβRII) and I (TβRI), vimentin, desmin, α-SMA, connective tissue growth factor (CTGF), Id2, and p21 (Figure 1), all representing known pro-fibrogenic matricellular components or mediators involved in hepatic fibrogenesis 23–25. These results partially confirm and extend previous findings demonstrating that LX-2 have attributes of primary human HSC in culture [4]. In addition, the expression of the tissue inhibitor of metalloproteinase-1 (TIMP-1) in LX-2 cells, as previously reported [4], was confirmed (not shown).

### Conventional and Molecular Karyotyping

**Figure 2:**
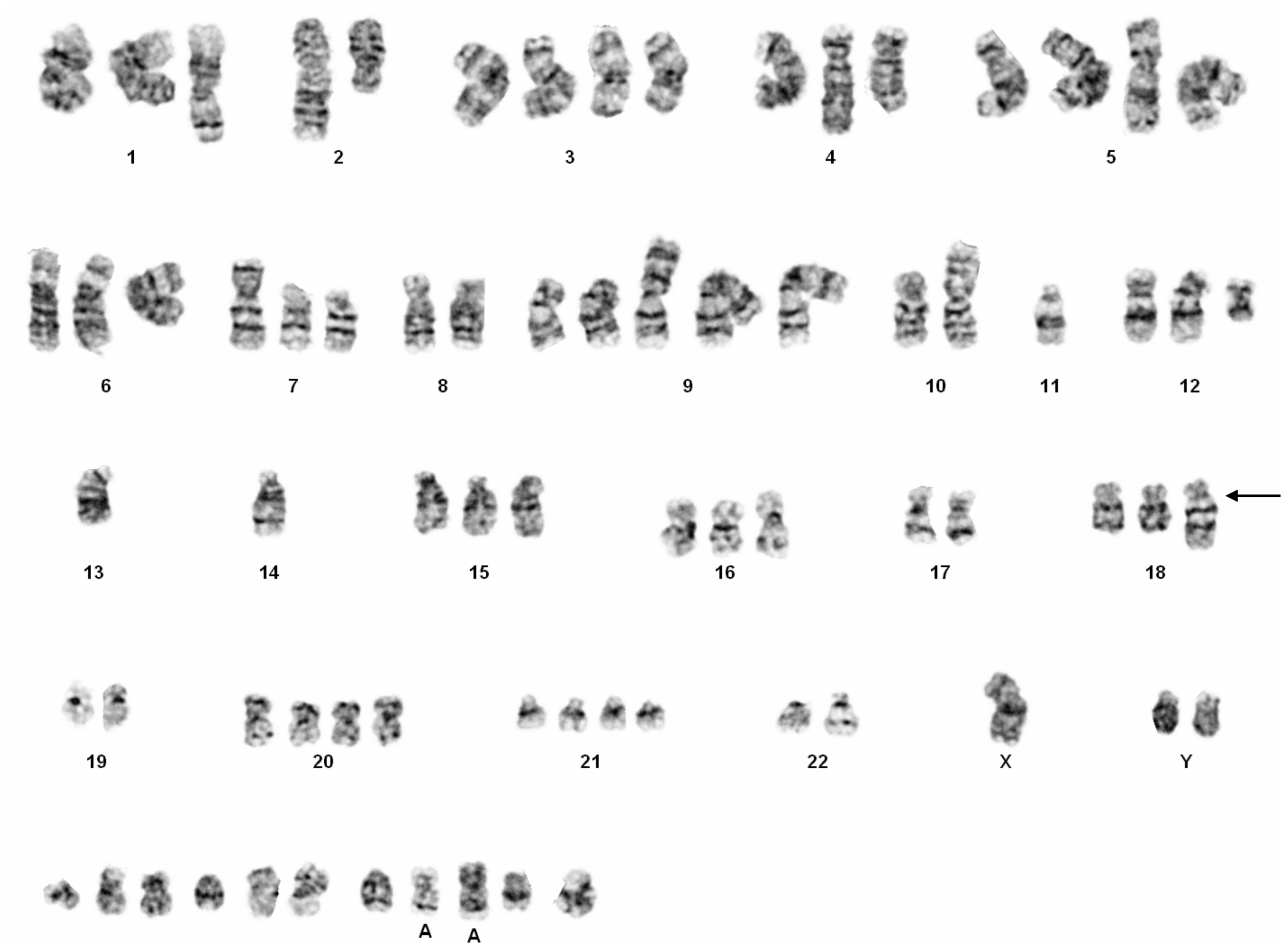
Karyogram of LX-2 cell. Representative numbers and appearance of chromosomes that was found in one LX-2 cell as obtained after Giemsa stain in lightmicroscopic analysis. The karyotype was determined to 74, XYY, +del(1)(q4?1), der(2;10)(p10;p10), +del(3)(q25)x2, +der(4)add(4)(q31), der(5)?t(5;9)(q35;q34)?del(5)(p15), der(5)add(5)(p1?3), +der(5)add(5)(p1?3)x2, +6,del(7)(p1?3), +del(7)(p1?3), +der(9)(11q23->11q21::14q32->14q11.2::9p13->9qter)x3, +i(10)(q10), −11, del(11)(p11.2), +del(12)(pter->q12::q15->q22::q24.2->qter), −13, −14, +15, +der(16)t(16;17)(q11.1;q11.2), +der(18)(18pter->18q11.2::1q25->1q32::7q22->7qter), +20, +20, +21, +21, der(22)?t(22;?)(q11.2;?), +11mar, respectively. Please note that a characteristic derivative of chromosome 18, i.e. +der(18)(18pter->18q11.2::1q25->1q32::7q22->7qter), is marked with an arrow in this figure. A characteristic derivative of chromosome 18 that contains part of chromosomes 1 and 7 was seen in three karyograms and is marked by a black arrow.

To characterize LX-2 genetically, we first performed chromosome G-banding analysis using different LX-2 cell cultures. This analysis revealed that LX-2 cells have several numerical and structural chromosomal aberrations (Figure 2). The karyotype as assessed by this methodology was described as: 55∼78, der(X)add(X)(p22.?1), Y [4], del(1)(q4?1) [2], +del(1)(q4?1) [3], der(2;10)(p10;p10) [4], +der(2;10)(p10;p10) [2], del(3)(q25) [3], +del(3)(q25)x2 [2], +der(3)add(3)(q2?7) [3], +der(3)?t(3;6)(p25;p2?3) [2], der(4)add(4)(q31) [4], +der(4)add(4)(q31) [2], +der(4)t(?1;4)(p36.1;q35) [3], der(5)?t(5;9)(q35;q34)?del(5)(p15) [5], der(5)add(5)(p1?3) [4], +der(5)add(5)(p1?3)x2 [2], +6 [3], del(7)(p1?3) [3], +del(7)(p1?3) [2], der(9)(11q23->11q21::14q32->14q11.2::9p13->9qter) [2], +der(9)(11q23->11q21::14q32->14q11.2::9p13->9qter) [2],i(10)(q10) [3], del(11)(p11.2) [3], +del(12)(pter->q12::q15->q22::q24.2->qter) [3], −13 [4], −14 [4], +15 [2], +15 [2], der(16)t(16;17)(q11.1;q11.2) [2], +der(16)t(16;17)(q11.1;q11.2) [2], +der(18)(18pter->18q11.2::1q25->1q32::7q22->7qter) [2], +19 [2], +20 [4], +20 [4], +20 [2], +21 [3], +8∼13mar [5][cp5], respectively.

In order to further characterize this complex pattern of alterations in more detail, we next performed spectral karyotyping (SKY) that is much more versatile and demanding than conventional chromosome analysis.

**Figure 3:**
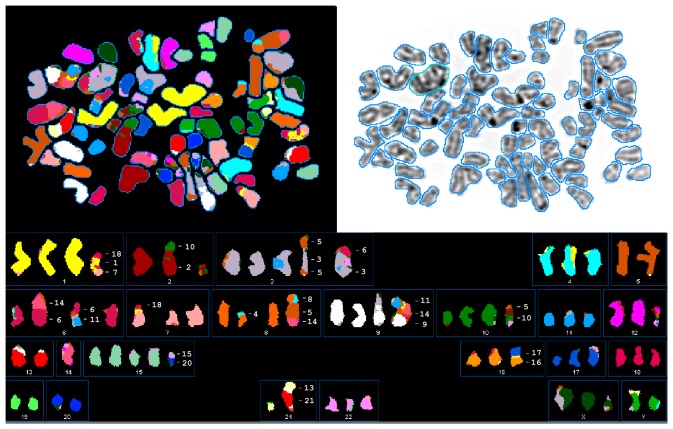
Spectral karyogram (SKY) of the hepatic stellate cell line LX-2. Shown is a representative image of LX-2 cells hybridized with a 24-color SKY probe (*upper left panel*). The chromosomes were counterstained with DAPI (*upper right panel*). The spectral images of hybridized metaphases were acquired using a cytogenetic workstation and grouped with the SkyView software (*lower panel*). Based on this analysis, the karyotype of this LX-2 metaphase is determined to: 73<3n+>, XXXYY, ish +der(1)t(1;18)(p11;q21)t(1;7)(q42;p12), der(2)t(2;10)(p11;q11), del(2)(q11), +der(3)t(3;6)(qter;q11), der(3)t(3;5)(p11;q23)t(3;5)(q24;q23)del(5)(q14), −5, +del(6)(p11), der(6)t(6;14)(p11;q11), der(6)t(6;11)(q11;q12), der(7)t(7;18)(p11;p11), del(7)(q21), +der(8)t(8;5)(q11;q11)t(5;14)(q31;q23), der(8)t(4;8)(q32;p12), +der(9)t(3;9)(p22;qter)del(9)(p11), der(9)t(9;14)(p12;q11)t(14;11)(q24;q14)del(11)(q12), +der(10)del(10)(p12)del(10)(q23), der(10)t(5;10)(p12;p11), del(11)(p14), der(11)del(11)(p14)del(11)(q23)x2, der(12)t(12;22)(q13;q12), −13, −14x2,+i(15)(p10), +der(15)t(15;20)(q15;q11), der(16)t(16;17)(p11;q11), del(17)(p11), −19, −20, −21, der(21)t(21;13)(p11;q11), del(X)(q24), del(X)(q25), del(X)(q21). Please note that the derivative that contains parts of chromosomes 1, 18 and 7 (cf. Figure 1) were also seen in this analysis.

**Figure 4:**
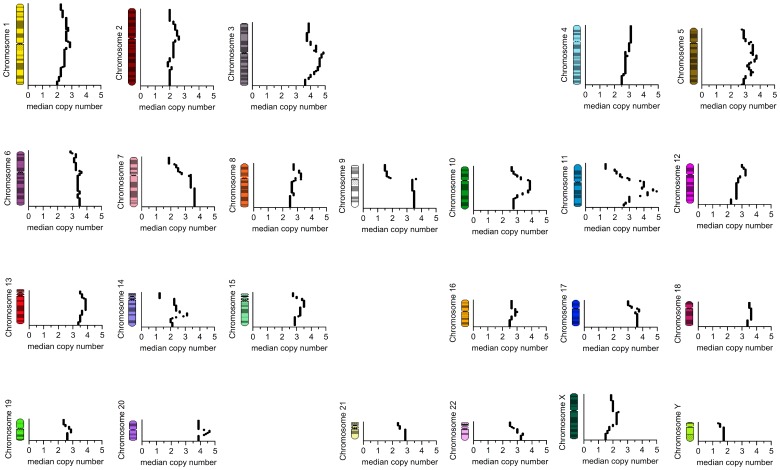
Median appearance of chromosome regions per cell based upon SKY analysis of eight metaphases from human LX-2 cell line. The average copy numbers per cell of each human chromosome region are shown in correspondence to their chromosome ideogram based on summary SKY analysis.

**Figure 5:**
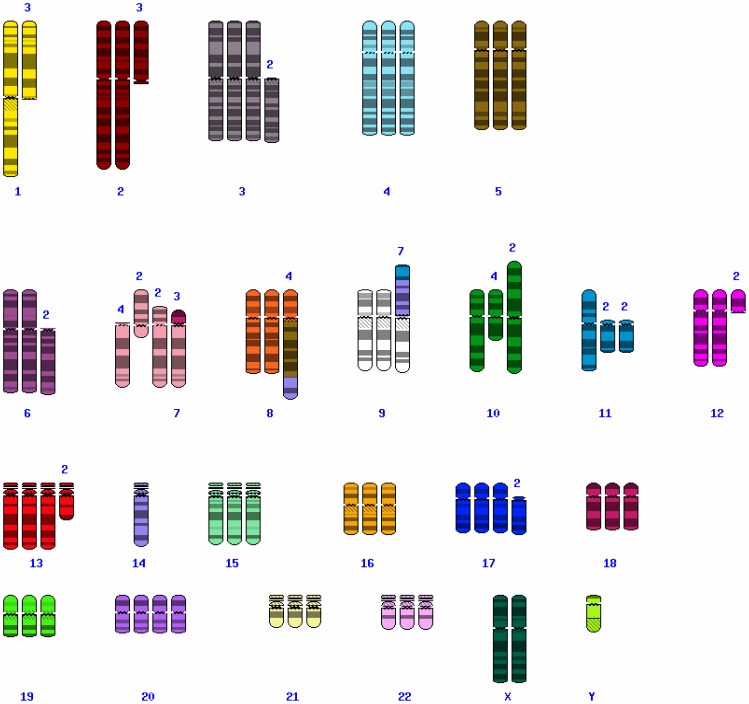
Complete SKYGRAM of LX-2 cells based on eight cells. Characters above rearranged chromosome ideograms are for numbers of metaphases with this aberration. Rearrangements recognized only once in SKY analysis are ignored. Based on this analysis, the complete SKYGRAM of LX-2 was determined to 64∼83<n3+/−>, XXY, −1, del(1)(q11) [3], del(2)(q11) [3], +del(3)(p11) [2], del(6)(p11) [2], del(7)(p11) [4], del(7)(q11) [2], del(7)(p15) [2], +der(7)t(7;18)(p11;p11) [3], der(8)t(5;8)(q11;q11)t(5;14)(q31;q23) [4], der(9)t(9;14)(p12;q11)t(11;14)(q14;q24)del(11)(q12) [7], del(10)(q22) [4],i(10)(q10) [2], der(11)del(11)(p11)del(11)(q22)x2 [2], del(12)(q11) [2], +del(13)(q21) [2],-14,-14, +del(17)(p11) [2],+20[cp8], respectively. More detailed SKYGRAM data for LX-2 cells were deposited in the SKY/M-FISH & CGH database located at the National Center for Biotechnology Information that can be found at: http://www.ncbi.nlm.nih.gov/sky/skyquery.cgi.

We detected more than 160 chromosomal rearrangements in eight different chromatograms by SKY indicating great genomic heterogeneity of this cell line (Table S2). However, several rearrangements between nonhomologous chromosomes were identified (Figure 3). Notably, translocation products der(9)t(9;11;14), and der(5)t(5;8;14) were frequently observed and especially der(9)t(9;11;14) was present in almost every analysed metaphase (Figure 4, Table S2). In the final SKYGRAM that displays the SKY data of all eight metaphases in the form of coloured chromosome ideograms, it becomes more clear that these chromosomal alterations mainly affect the loss of arm segments in 1q, 2p, 7p, 10q and 14q or rearrangement of individual chromosomes, i.e. der(9)t(9;11;14) and der(8)t(5;8;11), respectively (Figure 5). Based on their common appearance, it is most likely that these rearrangements have already occurred during onset and/or early propagation of this cell line and that they are relatively stable because we observed them in different cell passages (not shown).

**Figure 6:**
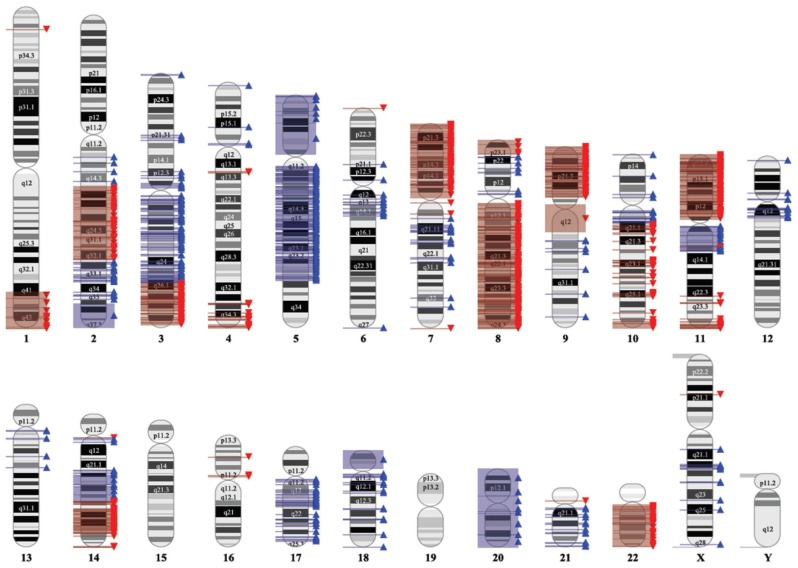
Chromosomal imbalances detected in the LX-2 cell line using a SNP 6.0 Array. Gains and losses are shown by red and blue triangles, respectively.

Subsequently, we performed whole-genome SNP array analysis to confirm the different chromosomal imbalances observed by SKY. This analysis is a useful tool for studying slight variations between whole genomes and provides a signature that is characteristic for each individual. Overall, the generated copy number profile substantially agrees with the described SKY data (cf. Figure 4 and 6). The comparison of results obtained in SKY (Figure 4) and SNP array analysis (Figure 6) indicates similar chromosomal gains in 3q, 5p, 5q, 11q, 14q, 17q, 20 and losses in 1q, 7p, 8q, 9p, and 11p. Therefore, the SKY results of the eight metaphases seem to reflect a representative intersection of the complete culture genome used for microarray analysis.

It is important to note that the LX-2 cell line was originally established from primary HSC that were transfected with the large SV40 T-antigen in passage 4 [4]. Subsequently, transfected cells were further passaged seven times before an individual clone (LX-1) was finally established. Afterwards LX-2 was expanded as a cellular subline that was able to grow under reduced serum conditions [4]. It might therefore be possible that these harsh selection conditions induced several independent subclonal rearrangements that were simultaneously passed during prolonged culturing and passages resulting in cellular heterogeneity. We presently do not known if the observed translocations or imbalances might be of relevance for the fibrogenic activities of LX-2 or if they are simply induced by the immortalization using the large T-antigen. In addition, SKY in our analysis does not allow concise molecular definition of the respective breakpoints to the gene level, so that we do not know if these rearrangements introduce alterations in gene expression or are causative for the establishment of the immortalized phenotype of this cell line.

#### STR analysis

Inter- and intraspecies cross-contamination in cell lines and misidentified cultures are a longstanding problem and a frequent cause of false experimental outcome and scientific misinterpretation. Although this is already recognized for over 60 years [26]–[31], urgent appeals to scientists to address this issue have not been widely accepted, including studies of the liver [32]. However, most cell lines (i.e. HeLa) that have already contributed for over 60,000 publications are still incompletely characterized [33]. Therefore, it was recently proposed that the unambiguous cell authentication in studies using cell lines should be a consideration during peer review of papers submitted for publication or during review of grants submitted for funding [34].

To address this problem, an international expert team of scientists has therefore prepared a consensus standard for the authentication of human cells using STR profiling strategies [35]. This approach provides a simple method for rapid and cheap cell line identification [35] and further enables the establishment of a public STR profile database under the auspices of the National Center for Biotechnology information [30].

Consistent with these recommendations, we next performed STR profiling for LX-2 cells and all other cell lines used in this study by using two different commercially available kit systems, As expected, both kits revealed a male genotype in LX-2 (Table S3). In addition, LX-2 were genotyped to be homozygous for the STR loci D16S539 (13), D7S820 (11), TH01 (9.3), vWA (17), D8S1179 (13), D2S1338 (17), D18S51 (12) and to be heterozygous for markers D13S317 (11, 13), D5S818 (11, 12), CSF1PO (10, 12), TPOX (8, 9), D21S11 (28, 31), D3S1358 (13, 15), D19S433 (13, 15.2), and FGA (21, 26), respectively (Table S3). A detailed STR matching analysis of this STR profile with respective online tools provided by the DSMZ or ATCC revealed that this STR profile is unique for LX-2 and not deposited yet in these data bases. The STR profiles for HepG2, Hep3B and Wi-38 were identical to those that are given in respective ATCC and DSMZ depositories (for details see Material and Methods).

Expression analysis further revealed that LX-2 cells express typical markers of HSC/MFB including fibronectin, collagen type IV, collagen type I, various TGF-β receptors (i.e. endoglin, TβRII, TβRI), vimentin, desmin, α-smooth muscle actin, CTGF, and p21 suggesting that this cell line is indeed originated from hepatic stellate cells (Figure 1).

It is presently not possible to predict the impact of the observed genetic alterations for studies performed in LX-2 cells. However, the observed chromosomal aberrations and the definition of a unique, characteristic STR profile for LX-2 will now help to fulfil the recommendation for cellular authentication required by granting agencies and scientific journals when working with LX-2 and to avoid unreliable experimental outcomes and scientific misinterpretation. Hopefully, our study increase the value and suitability of LX-2 cells in liver research and provides an important benchmark for proper genetic characterization of widely used cell lines in the field of hepatology.

## Supporting Information

Figure S1 **Usage of LX-2 cells during 2003–2013.** Studies using LX-2 cells were identified in a PubMed search for “LX-2” and “LX2”. Please note the increasing number of reports using LX-2 cells during recent years.
(PDF)

Figure S2 **Light microscopic appearance of LX-2.** Cells were seeded in cell culture dishes and representative images taken from cultures at **(A)** high and **(B)** low densities. Original magnifications are 200× (A) and 400 (B), respectively. Space bars in each figure represents 50 µm.
(PDF)

Figure S3 **LX-2 immunocytochemistry. (A–C)** LX-2 cells were incubated with an Alexa Fluor ® 488 phalloidin conjugate and nuclei were stained with DAPI. The cells were analysed by UV microscopy for the Alexa dye **(A)** and DAPI **(B)**. The overlay of **(A)** and **(B)** is shown in **(C)**. **(D–F)** LX-2 cells were stained with an antibody specific for α-SMA **(D)** and nuclei stained with DAPI **(E)**. The overlay is shown in **(F)**. **(G–I)** A stain with an unspecific mouse IgG served as an internal control for antibody specificity. **(J–L)** LX-2 cells were permeabilized and stained with a polyclonal antibody raised against amino acids 4–30 mapping near the N-terminus of large SV40 T Ag **(J)** and nuclei stained with DAPI **(K)**. The overlay of **(J)** and **(K)** is shown in **(L)**. **(M–O)** A stain with an unspecific control rabbit IgG in this analysis served to demonstrate antibody specificity **(E)**. Original magnifications are 400x **(A–C, G–O)** and 200 **(D–F)**, respectively. Space bars in each figure represents 50 µm.
(PDF)

Table S1 **Antibodies used in this study.**
(PDF)

Table S2 **Spectral karyotyping (SKY) analysis of LX-2 cells.**
(PDF)

Table S3 **Short tandem repeat analysis of cells used in this study.**
(PDF)
