# Supplementary figures and images for: Genetic Characteristics of the Human Hepatic Stellate Cell Line LX-2
Source: PLoS One. 2013 Oct 8;8(10):e75692. doi: 10.1371/journal.pone.0075692 (PMC3792989; doi:10.1371/journal.pone.0075692)

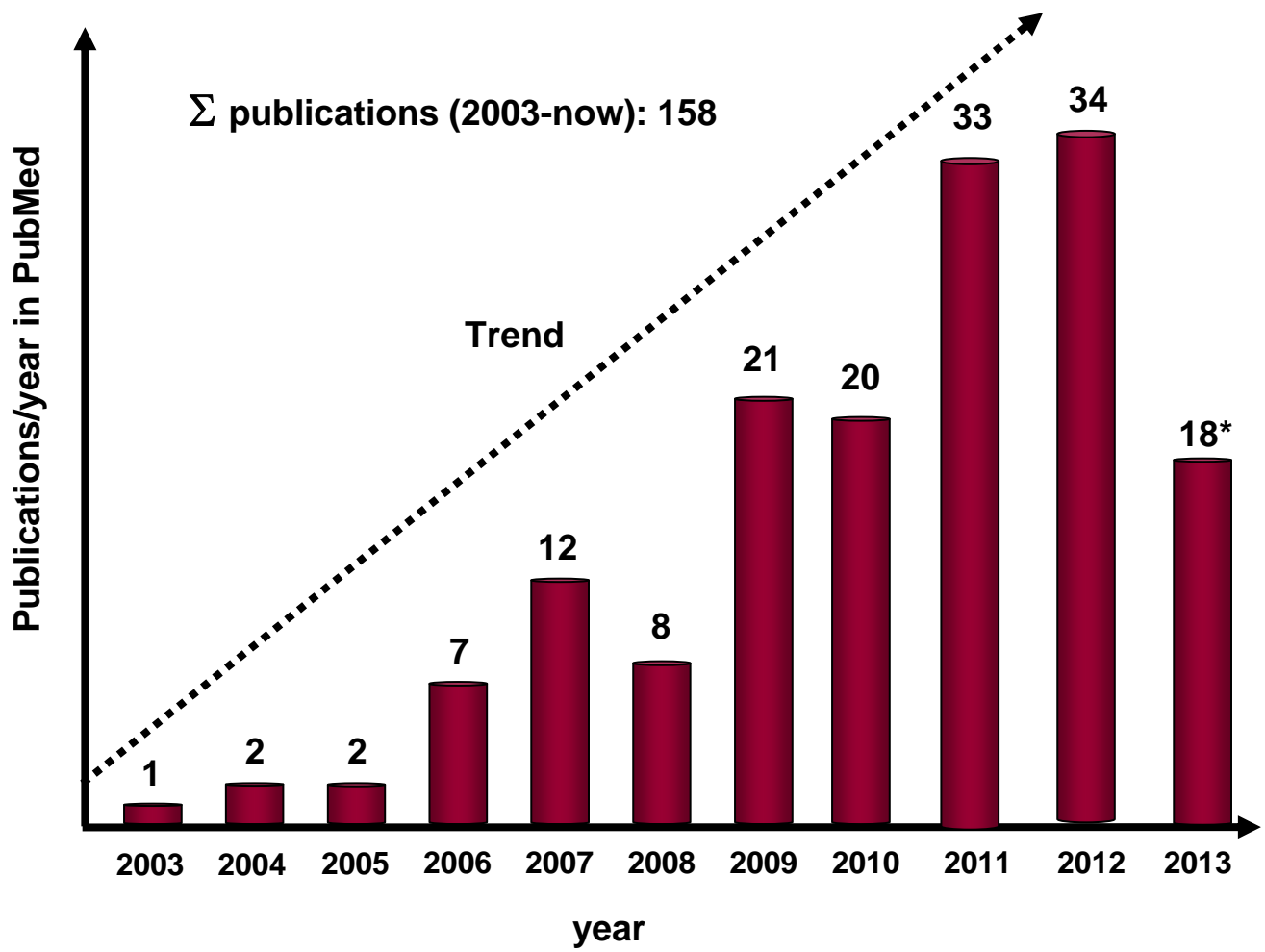

\* only first four months of year 2013

Weiskirchen *et al.*, Suppl. Figure 1

Supplement: Figure S1 — Usage of LX-2 cells during 2003–2013. Studies using LX-2 cells were identified in a PubMed search for “LX-2” and “LX2”. Please note the increasing number of reports using LX-2 cells during recent years. (PDF) [file pone.0075692.s001.pdf]

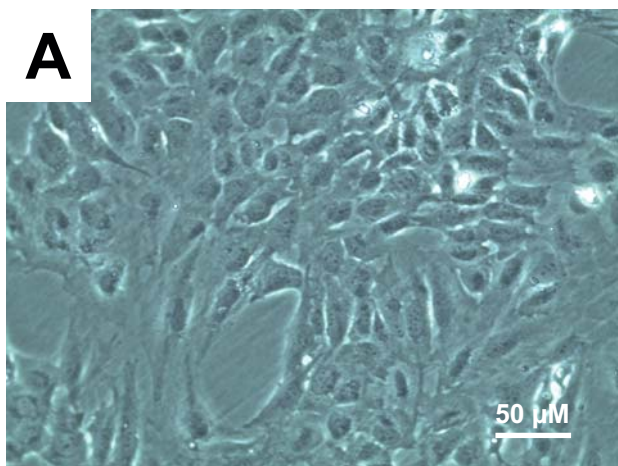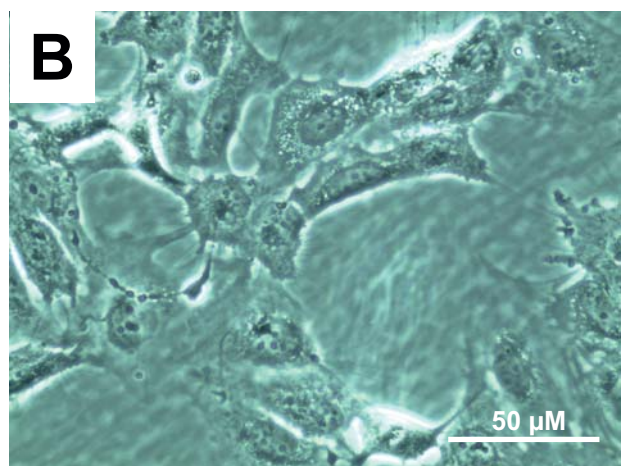

**Weiskirchen *et al.*, Suppl. Figure 2**

Supplement: Figure S2 — Light microscopic appearance of LX-2. Cells were seeded in cell culture dishes and representative images taken from cultures at (A) high and (B) low densities. Original magnifications are 200× (A) and 400 (B), respectively. Space bars in each figure represents 50 µm. (PDF) [file pone.0075692.s002.pdf]

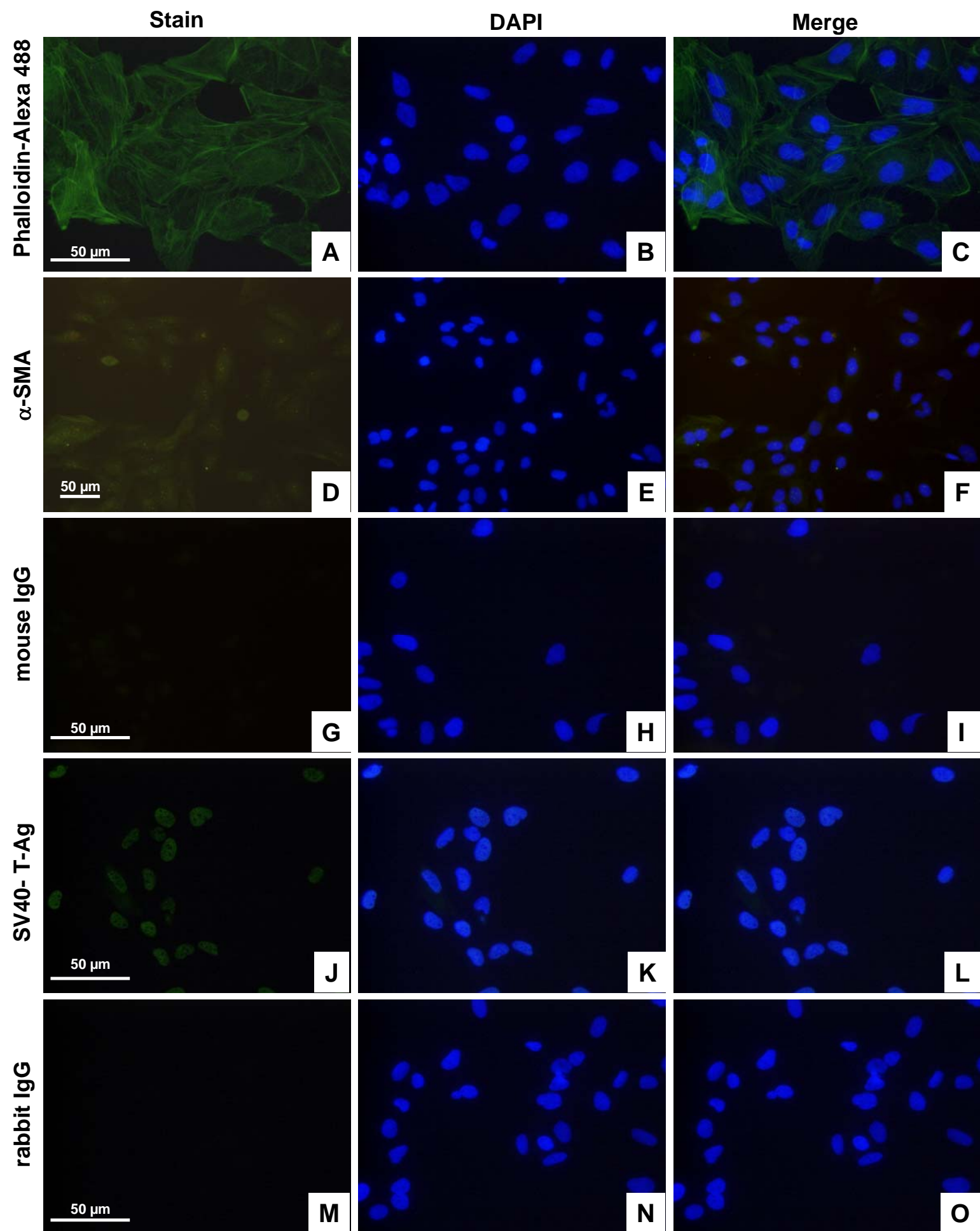

Supplement: Figure S3 — LX-2 immunocytochemistry. (A–C) LX-2 cells were incubated with an Alexa Fluor ® 488 phalloidin conjugate and nuclei were stained with DAPI. The cells were analysed by UV microscopy for the Alexa dye (A) and DAPI (B). The overlay of (A) and (B) is shown in (C). (D–F) LX-2 cells were stained with an antibody specific for α-SMA (D) and nuclei stained with DAPI (E). The overlay is shown in (F). (G–I) A stain with an unspecific mouse IgG served as an internal control for antibody specificity. (J–L) LX-2 cells were permeabilized and stained with a polyclonal antibody raised against amino acids 4–30 mapping near the N-terminus of large SV40 T Ag (J) and nuclei stained with DAPI (K). The overlay of (J) and (K) is shown in (L). (M–O) A stain with an unspecific control rabbit IgG in this analysis served to demonstrate antibody specificity (E). Original magnifications are 400x (A–C, G–O) and 200 (D–F), respectively. Space bars in each figure represents 50 µm. (PDF) [file pone.0075692.s003.pdf]
