# Supplementary material for: Genetic Characteristics of the Human Hepatic Stellate Cell Line LX-2
Source: PLoS One. 2013 Oct 8;8(10):e75692. doi: 10.1371/journal.pone.0075692 (PMC3792989; doi:10.1371/journal.pone.0075692)
Supplement: Table S1 — Antibodies used in this study. (PDF) [file pone.0075692.s004.pdf]

Supplementary Table 1:

Antibodies used in this study

Antibody	clonality	Supplier	Epitope location	Species	Dilution
**TGF-β receptors**					
sc-398	Poly	Santa Cruz Biotech., Santa Cruz, CA, USA	peptide mapping within a C-terminal cytoplasmic domain of human TGFβRI	h, m, r	1 : 500
sc-400	Poly	Santa Cruz	peptide mapping within a cytoplasmic domain of human TGFβRII	h, m, r	1 : 1,000
sc-6199	Poly	Santa Cruz	raised against a peptide mapping at the C-terminus of human TGFβRIII	h, m, r	1 : 500
**target proteins**					
556430	Mono	BD Pharmingen, Heidelberg, Germany	recognized epitope maps to residues 145-164 of human p21	h, m, r	1 :1,000
sc-14939	Poly	Santa Cruz	CTGF, recognizes internal region of human CTGF	h, m, r	1 : 1,000
ab-92547	Mono	abcam, Cambridge, UK	raised against a synthetic peptide corresponding to the C-terminus of human Vimentin	h, m, r	1 : 1,000
sc-489	Poly	Santa Cruz	Id2, epitope mapping to the C-terminus, mouse origin	h, m, r	1 : 500
PS065	Poly	Monosan, Hycultec GmbH, Beutels-bach, Germany	rat Collagen I	h, m, r	1 : 500
ab-6586	Poly	abcam, Cambridge, UK	human Collagen IV	h	1 : 1,000
AB1954	Poly	Millipore, Billerica, MA, USA	purified rat plasma fibronectin	m, r	1 : 1,000
sc-7559	Poly	Santa Cruz	epitope mapping near the C-terminus of human Desmin	h, m, r	1 : 500
sc-20800	Poly	Santa Cruz	amino acids 4-30 of the SV40 large T antigen	SV40 large T antigen	1 : 1,000
CBL 171	Mono	Millipore	peptide corresponding to the ten N-terminal amino acids of the α-smooth muscle isoform of actin (clone ASM-1)	h, m, r	1 : 1,000
**protein loading**					
A5441	Mono	Sigma-Aldrich, Taufkirchen, Germany	β-actin, raised against a synthetic KHL-coupled slightly modified peptide raised against β-cytoplasmic actin N-terminal peptide (clone AC-15)	h, m, r	1 : 10,000
