# Supplementary material for: Genetic Characteristics of the Human Hepatic Stellate Cell Line LX-2
Source: PLoS One. 2013 Oct 8;8(10):e75692. doi: 10.1371/journal.pone.0075692 (PMC3792989; doi:10.1371/journal.pone.0075692)
Supplement: Table S2 — Spectral karyotyping (SKY) analysis of LX-2 cells. (PDF) [file pone.0075692.s005.pdf]

Supplementary Table 2:

Spectral karyotyping (SKY) analysis of LX-2 cells

Abberation	P02	P04	P07	P08	P10	P11	P12	P13	total no. chromosomes	pos. cells	%
der(1)t(1;6)(q41;q22.3)del(1)(p35)	1								1	1	12,5
der(1)t(1;6)(q22;q24)		1							1	1	
der(1)t(1;7)(p22;q22)						1			1	1	
der(1)t(1;7)(p32;q22)t(1;11)(q12;?)	1								1	1	
der(1)t(1;7)(q23;q22)del(1)(p22)		1							1	1	
der(1)t(1;18)(p11;q21)t(1;7)(q42;p12)							1		1	1	
der(1)del(1)(p31)del(1)(q12)	1								1	1	
der(2)t(2;10)(p11;q11)							1		1	1	
del(1)(p11)					1				1	1	
del(1)(q31)			1						1	1	
del(2)(p11)						1			1	1	
del(2)(q11)	1					1	1		3	3	37,5
der(2)del(2)(p21)del(2)(q23)					1				1	1	
der(2)del(2)(p16)del(2)(q32)	1								1	1	
der(2)del(2)(p13)del(2)(q11)	1								1	1	
der(2)del(2)(p11)del(2)(q23)					1				1	1	
der(2)del(2)(p11)del(2)(q24)			1						1	1	
der(3)t(5;3)(q22;p12)t(3;5)(q24;q23)					1				1	1	
der(3)t(3;20)(q26;q11)				1					1	1	
der(3)t(3;5)(p14;q23)					1				1	1	
der(3)t(3;5)(p11;q23)t(3;5)(q24;q23)del(5)(q14)							1		1	1	
der(3)t(3;6)(qter;q11)							1		1	1	
del(3)(q11)	1								1	1	
del(3)(p11)	1	2							3	2	
del(3)(q13)		1							1	1	
del(3)(p13)					2				2	1	
del(3)(p14)		1							1	1	
del(3)(q22)						1			1	1	
del(3)(q25)						2			2	1	
del(3)(q26)								1	1	1	
der(3)del(3)(p12)del(3)(q26)								1	1	1	

del(4)(q21)				2					2	1	
del(4)(q23)						1			1	1	
del(4)(q31)		1							1	1	
del(4)(q11)		1							1	1	
der(4)t(4;5)(q31;q23)						1			1	1	
der(5)t(5;14)(q33;q23)		1							1	1	
der(5)t(5;5)(q31;p15)					1				1	1	
der(5)t(11;5)(q11;p13)			1						1	1	
der(5)t(5;11)(q14;q14)				1					1	1	
Dup(5)(q35q31)				1					1	1	
del(5)(p11)		1							1	1	
del(5)(q15)				1					1	1	
del(5)(q21)		1			1				2	2	
del(5)(q22)						2			2	1	
del(5)(p12)	1								1	1	
inv(5)(p15q31)x2,del(6)(q11)								1	1	1	
der(6)t(6;11)(q11;q12)							1		1	1	
der(6)t(6;14)(p11;q11)							1		1	1	
der(6)del(6)(p21)del(6)(q12)		1							1	1	
del(6)(p11)		1					1		2	2	
del(6)(p24)					1				1	1	
del(6)(q22)						1			1	1	
del(7)(p11)	1	1	1						3	3	37,5
del(7)(p14)				1					1	1	
del(7)(p15)					1			1	2	2	
del(6)(p23)			3						3	1	
del(7)(p15)		1							1	1	
del(7)(q11)	1	1							2	2	
del(7)(q21)							1		1	1	
der(7)t(1;7)(q36;p12)del(1)(p22),del(7)(p11)						1			1	1	
der(7)t(7;20)(p11;p11)						1			1	1	
der(7)t(18;7)(p11;p11)		1	1				1		3	3	37,5
der(8)t(4;8)(q32;p12)							1		1	1	
der(8)t(8;5)(q11;q11)t(5;14)(q31;q23)					1	1	1	1	4	4	50
der(8)t(8;14)(q22;q22)				2					2	2	
del(8)(p11)	1								1	1	

del(8)(p21)			1			1			2	2	
i(8)(q10)	1								1	1	
der(8)del(8)(p21)del(8)(q11)			1						1	1	
del(8)(p11p21)	1								1	1	
del(8)(q11)		1							1	1	
del(8)(p12)	1		1						2	2	
del(9)(p11)	2								2	1	
del(9)(p12)				1					1	1	
del(9)(p?)	1								1	1	
del(9)(p21)			1						1	1	
del(9)(q11)		1							1	1	
der(9)t(9;3)(qter;p22)del(9)(p11)							1		1	1	
der(9)t(9;Y)(p11;p11)				1					1	1	
der(9)t(9;14)(p12;q11)t(11;14)(q14;q24)del(11)(q12)	1	2	1		2	1	1	2	10	7	87,5
der(10)t(5;10)(p12;p11)							1		1	1	
der(10)del(10)(p11)del(10)(q23)			1						1	1	
der(10)del(10)(p12)del(10)(q23)							1		1	1	
der(10)del(10)(p13)del(10)(q21)					1				1	1	
der(10)del(10)(p14)del(10)(q22)								1	1	1	
del(10)(p11)								1	1	1	
del(10)(p12)			1						1	1	
del(10)(q11)		1							1	1	
del(10)(q22)	1			3		2		1	7	4	50
del(10)(q24)								1	1	1	
i(10)(q10)	1				1				1	1	
del(11)(p13)		2							2	1	
del(11)(p14)							1		1	1	
del(11)(q13)				1					1	1	
del(11)(q22)					1				1	1	
der(11)del(11)(p11)del(11)(q14)				1					1	1	
der(11)del(11)(p11)del(11)(q22)	2							2	4	2	
der(11)del(11)(p12)del(11)(q14)			2						2	1	
der(11)del(11)(p14)del(11)(q23)							2		2	1	
der(11)t(11;5)(q14;q14)				1					1	1	
der(11)t(11;14)(p11;q32)del(11)(q22)	1								1	1	
der(11)t(11;22)(p11;q12)del(11)(q22)								2	2	1	

der(11)del(11)(p14)del(11)(q22)	1					1			2	2	
i(11)(q10)						1			1	1	
der(12)t(12;22)(q13;q12)							1		1	1	
del(12)(q11)		2	1						3	2	
del(12)(p12)			1						1	1	
del(12)(p12.3)								3	3	1	
del(13)(q21)			1	1					2	2	
del(13)(q22)					1				1	1	
del(13)(q32)		1							1	1	
der(13)t(13;15)(p11;q11)								1	1	1	
der(13)t(20;13)(q11;pter)			1						1	1	
der(14)t(21;14)(q11;pter)			1						1	1	
i(15)(p10)							1		1	1	
i(15)(q10)								1	1	1	
del(15)(q23)		2							2	1	
del(15)(q15)					1				1	1	
der(15)t(3;15)(q11;p11)				1					1	1	
der(15)t(8;15)(p11;pter)					1				1	1	
der(15)t(15;13)(qter;q12)			1						1	1	
der(15)t(15;20)(q15;q11)							1		1	1	
der(16)t(16;17)(p11;q11)							1		1	1	
der(16)t(16;17)(q21;q21)		1							1	1	
del(16)(p11)				1		1			2	2	
idic(16)(pter)del(16)(q12)del(16)(q12)					1				1	1	
der(17)del(17)(p?)del(17)(q?)			1						1	1	
der(17)del(17)(p11)del(17)(q12)					1				1	1	
der(17)t(17;21)(qter;q11)			1						1	1	
del(17)(p11)		1					1		2	2	
del(17)(q21)								1	1	1	
i(17)(q10)		1							1	1	
del(18)(p11)		1							1	1	
del(18)(q12)					1				1	1	
der(18)t(3;18)(p13;pter)								1	1	1	
der(18)t(8;18)(p11;pter)								1	1	1	
der(18)t(16;18)(p11;p11)			1						1	1	
der(18)dup(18)(q11q21)dup(18)(q11q21)				1					1	1	

der(19)t(7;19)(q11;p12)					1				1	1	
del(19)(p12)						1			1	1	
del(19)(p13.1)		1							1	1	
del(19)(q13.1)				2					2	1	
der(20)t(20;9)(q11;q22)						1			1	1	
der(20)t(20;Y)(p11;?)	1								1	1	
del(20)(p11)		2							2	1	
del(20)(q12)				4					4	1	
der(21)t(21;13)(p11;q11)							1		1	1	
der(22)t(22;11)(p11;q11)del(11)(q14)						1			1	1	
der(22)t(22)(8;22)(q22;p11)t(22)(19;22)(q12;q12)						1			1	1	
der(X)del(X)(p21)del(X)(q21)					1				1	1	
der(X)t(X;2)(q22;q32)			2						2	1	
der(X)t(2;X)(p11;q11)								1	1	1	
der(X)t(X;10)(q28;q22)del(X)(p11)				1					1	1	
del(X)(q21)							1		1	1	
del(X)(q24)							1		1	1	
del(X)(q25)							1		1	1	
i(X)(q10)								1	1	1	
der(Y)t(3;Y)(p12;p11)		1							1	1	
der(Y)t(21;Y)(q11;p11)						1			1	1	

Note: Chromosome aberrations that were found in 3 or 4 cells of all chromatograms analysed (n = 8) are marked in light grey and those obtained in 7 of 8 cells (87.5%) in dark grey.
