# Supplementary material for: Genetic Characteristics of the Human Hepatic Stellate Cell Line LX-2
Source: PLoS One. 2013 Oct 8;8(10):e75692. doi: 10.1371/journal.pone.0075692 (PMC3792989; doi:10.1371/journal.pone.0075692)
Supplement: Table S3 — Short tandem repeat analysis of cells used in this study. (PDF) [file pone.0075692.s006.pdf]

Supplementary Table 3:

Short tandem repeat analysis of cells used in this study

STR Locus	LX-2	HepG2	Hep3B	Wi-38 (VA-13)
D16S539	13	12; 13	10	11; 12
D7S820	11	10	8; 10	9; 11
D13S317	11; 13	9; 13	12; 14	11
D5S818	11; 12	11; 12	13	10
CSF1PO	10; 12	10; 11	8	10; 12
TPOX	8; 9	8; 9	9	8
Amelogenin	X; Y	X; Y	X	X
TH01	9.3	9	6; 7	9.3
vWA	17	17	17	19; 20
D8S1179	13	15; 16	12	14
D21S11	28; 31	29; 31	30; 31	30; 30.2
D3S1358	13; 15	15; 16	15	16; 17
D2S1338	17	19; 20	21; 25	19; 25
D19S433	13; 15.2	15.2	12.2; 14	13; 16.2
D18S51	12	13; 14	20	16; 18
FGA	21; 26	22; 25	18	22; 24

* Note: STR loci of LX-2 were genotyped by usage of the PowerPlex® 1.2 System and the AmpFlSTR® Identifiler® PCR Amplification Kit. All other cell lines were genotyped by use of the AmpFlSTR® Identifiler® PCR Amplification Kit. These preformatted kit systems provide all of the materials necessary for coamplification of nine (PowerPlex® 1.2. System) or 15 STR loci (AmpFlSTR Identifiler® PCR Amplification Kit). In both kit systems, one of the amplified markers corresponds to the sex identification locus Amelogenin that display different sizes when amplified from X- or Y-chromosomes, thereby allowing gender-determination.
